# Linear Viscoelasticity of Human Ocular Tissues During Tensile Stress Relaxation

**DOI:** 10.1167/iovs.67.5.26

**Published:** 2026-05-13

**Authors:** Somaye Jafari, Atharva Shetye, Joseph L. Demer

**Affiliations:** 1 Department of Ophthalmology, Stein Eye Institute, Los Angeles, California, United States; 2 Mechanical and Aerospace Engineering Department, University of California, Los Angeles, California, United States; 3 Neuroscience Interdepartmental Program, University of California, Los Angeles, California, United States; 4 Department of Neurology, University of California, Los Angeles, California, United States; 5 Department of Bioengineering, University of California, Los Angeles, California, United States

**Keywords:** optic nerve, Prony series, sclera, stress relaxation, viscoelasticity

## Abstract

**Purpose:**

To quantitatively describe viscoelastic properties, we characterized the tensile stress relaxation of human ocular tissues using a Prony series model.

**Methods:**

Specimens from eight pairs of postmortem human eyes were dissected from six regions: the anterior, equatorial, posterior, and peripapillary sclera; the optic nerve (ON); and the optic nerve sheath (ONS). Each specimen underwent uniaxial tensile loading under controlled physiological conditions at strain levels ranging from 4% to 6% to identify the optimal strain range within which the tissues exhibit linear viscoelastic behavior. Stress relaxation curves were fitted to a generalized Maxwell model using a Prony series to determine tissue-specific relaxation time constants and relative moduli.

**Results:**

All tissues exhibited linear viscoelastic behavior within 5% strain. The anterior sclera showed the greatest stress level, with 12.6 MPa instantaneous modulus and 8.8 MPa equilibrium modulus, whereas the ON exhibited the fastest stress decay and lowest stiffness, with moduli of 3.5 MPa and 1.1 MPa, respectively. The ON had the longest long-term relaxation time of 460 ± 77 seconds, and the ONS had the shortest time at 60 ± 5 seconds. Prony series parameters successfully captured the relaxation profiles across all tissues.

**Conclusions:**

This study supports the use of Prony-based models for numerical simulation to describe the region-specific viscoelasticity of ocular tissues. These findings provide foundational data for future investigations into ocular biomechanics, particularly under dynamic or pathologic loading.

Ocular saccade speed surpasses movements of all other body parts, increasing with rotation amplitude to ∼500°/s for a 40° saccade occurring in only 100 ms.^1^ While globe inertia is negligible, release experiments show that the oculomotor plant, comprising the globe, extraocular muscles, and surrounding orbital tissues, exhibits an effective rotational elasticity of ∼0.25 gm/°. The dynamic response of this overall system is characterized by two dominant time constants, approximately 20 ms and 1.0 s, reflecting fast and slow components of orbital tissue viscoelasticity.^1^ During saccades, a major load on the eye arises as a result of the viscous resistance of the relaxing antagonist extraocular muscle, which generates force opposing rapid eye rotation.^1^ Extraocular muscle (EOM) forces during saccades appreciably exceed forces during static fixations,^1^ transiently doubling the ultimate static force that is required to maintain eccentric rotational position.^2^ This suggests that ocular strains associated with saccades markedly exceed the static case and could contribute to optic nerve (ON) and peripapillary retinal disease.

Viscoelastic properties of ocular tissues are critical to understanding their behavior during rapid eye movements and can be characterized by creep and stress relaxation experiments.^3^ Elastic and viscoelastic properties of the sclera have been extensively investigated using tensile testing, inflation testing, and indentation-based approaches, revealing pronounced regional variations in stiffness and time-dependent behavior, as comprehensively reviewed by Boote et al.^4^ Early uniaxial studies of peripapillary sclera in rabbit and monkey demonstrated rapid stress relaxation and quantified instantaneous and long-term viscoelastic moduli at low strains.^5^^,^^6^ Complementary whole-globe and posterior scleral inflation studies have shown that the sclera exhibits nonlinear, rate-dependent, and creep behavior under physiologic and elevated intraocular pressures, with distinct responses in the mid-posterior and peripapillary regions.^7^^,^^8^ Creep data exist for human sclera and cornea,^9^^–^^11^ chicken sclera,^12^ and bovine extraocular muscles^3^ and sclera.^13^ Importantly, scleral viscoelastic behavior has been shown to change in myopia, with increased creep rates reported in posterior and equatorial sclera from myopic chick and tree shrew eyes, implicating altered time-dependent material behavior in axial elongation.^9^^,^^14^

However, whole-eye inflation testing is unsuitable for isolating the ON and its sheath. The only ocular viscoelastic parameter currently assessed clinically is corneal hysteresis, whose physical basis remains unclear and whose association with disease is largely statistical.^15^ Our earlier creep study remains the only direct viscoelastic characterization of the human ON and its sheath.^16^ Stress relaxation testing captures stress decay under constant deformation and enables characterization of time-dependent viscoelastic behavior in ocular tissues.

While previous studies in ocular biomechanics have employed diverse constitutive modeling strategies addressing different mechanical behaviors, these approaches are not directly comparable. For example, Safa et al.^17^ modeled the porcine optic nerve head using a biphasic formulation with a viscoelastic solid matrix to capture fluid–solid interactions, while Whitford et al.^18^ developed an anisotropic hyperelastic model to describe the nonlinear elastic behavior of the human cornea. Corneal viscoelasticity has also been characterized using nonlinear viscoelastic frameworks, including quasi-linear viscoelastic formulations that combine a deformation-dependent elastic response with a time-dependent relaxation kernel^19^ and finite-deformation anisotropic viscoelastic models represented by rheological networks of equilibrium and nonequilibrium elements.^20^ By contrast, the Prony series provides a linear viscoelastic representation appropriate for modeling stress relaxation under small-strain conditions and has been successfully applied to a wide range of soft tissues, including nonocular biological materials,^21^^–^^23^ porcine cornea,^24^ and human ocular tissues via indentation^25^ and rheological testing.^26^ The modularity of the Prony series and its compatibility with finite element methods make it well suited for quantifying regional differences in ocular tissue viscoelasticity.

By characterizing stress relaxation in human ocular regions, including the ON, ON sheath, peripapillary sclera, and anterior, equatorial, and posterior sclera, we aimed to quantify regional variability in viscoelastic behavior. These insights are critical for refining computational models of ocular biomechanics and for understanding tissue load distribution in ocular health and disease.^27^^–^^29^

## Methods

### Specimen Preparation

Eight pairs of postmortem Caucasian eyes with long attached ONs from four male and four female donors with a mean ± SD age of 82 ± 9 years were obtained from the Lions Gift of Sight Eye Bank (Saint Paul, MN, USA) and air shipped to the laboratory. At the time of death, donors had been diagnosed with multiple diseases, including Alzheimer's disease, multiple organ failure, or cancer, making specific causes of death typically indeterminate. Experiments were conducted 24 to 48 hours postmortem and complied with ARVO Best Practices for Using Human Eye Tissue in Research.

**Figure 1.**
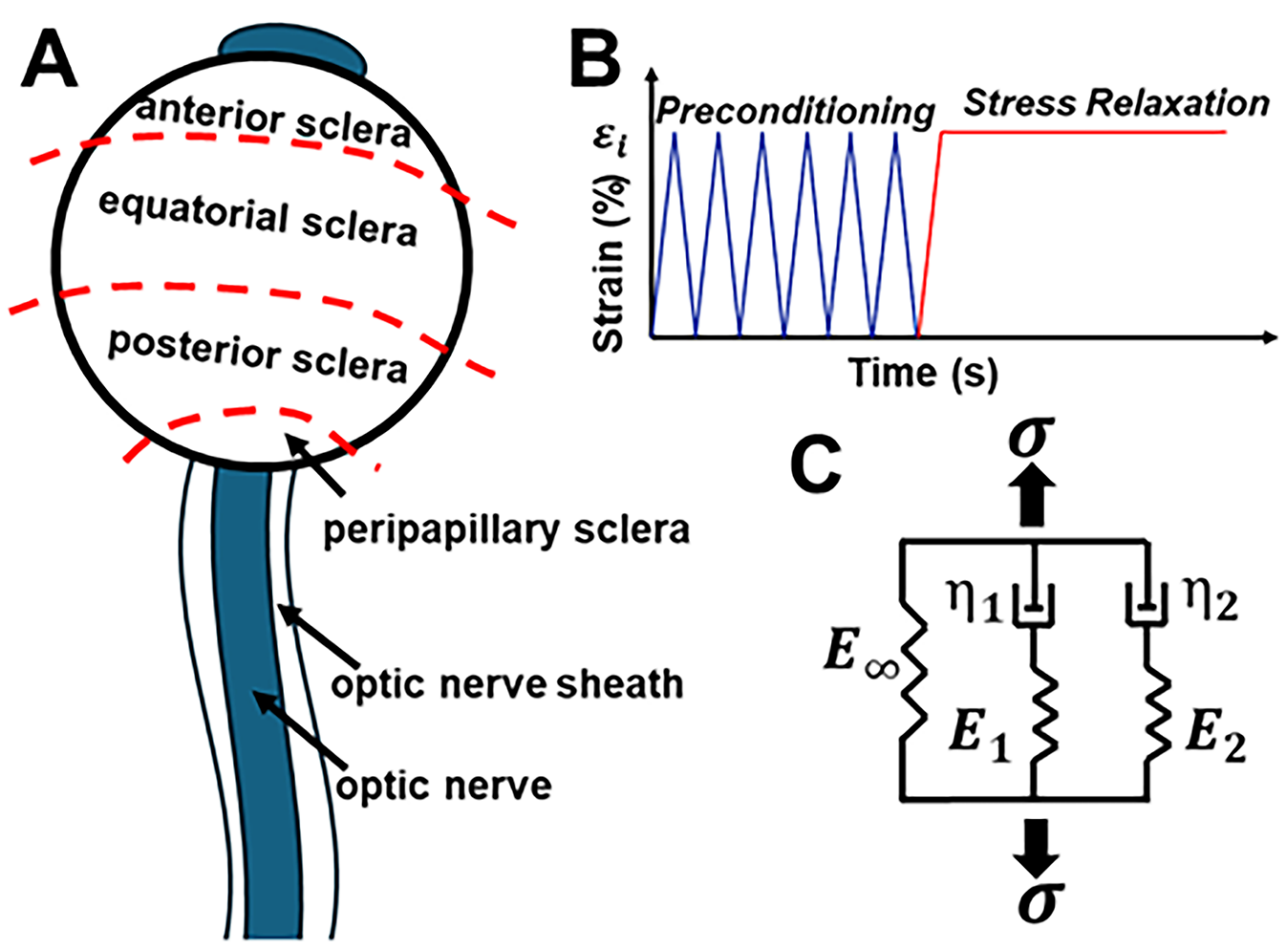
(**A**) Specimen regions. PPS was trephined from the annular region surrounding the optic nerve head. (**B**) Preconditioning and stress–relaxation loading. (**C**) Generalized Maxwell model implemented as second-order Prony series.

After eye bank harvesting, each eye was wrapped in gauze soaked in Ringer's lactate solution (B. Braun Medical, Bethlehem, PA, USA) and transported on ice without freezing. Upon arrival, specimens were continuously moisturized with Ringer's lactate solution to prevent dehydration. Scleral and optic nerve sheath (ONS) tissues were dissected into rectangular specimens excised tangentially from defined anatomic regions, as illustrated in Figure 1A. Anterior, equatorial, and posterior scleral specimens were obtained based on circumferential location relative to the limbus and posterior pole. Peripapillary sclera (PPS) was prepared as an annular region surrounding the ON head by trephining a 10-mm diameter disc centered on the optic nerve head and removing the nerve head using an 8-mm diameter trephine; the resulting scleral annulus was divided into equal circumferential segments. Although the peripapillary sclera is known to exhibit strong circumferential microstructural anisotropy near the ON head,^30^ the trephine-based preparation used here sampled a peripapillary/posterior scleral region extending modestly beyond the highly aligned collagen ring, resulting in specimens that reflect direction-averaged viscoelastic behavior. The ON was tested in its native cylindrical geometry.

Scleral specimens were dissected in both circumferential and radial directions, and ONS specimens were prepared in longitudinal and circumferential directions, as previously published.^31^ The dimensions and number of specimens are summarized in Table 1. Specimen thickness was measured using a digital caliper immediately prior to testing. Because scleral and ONS tissues are compliant and may have thickened with hydration, the reported values represent an effective thickness under experimental handling conditions and may overestimate the true tissue thickness. To prevent slippage during loading, the ends of each specimen were glued to cardboard using cyanoacrylate and clamped in the testing apparatus. Specimen lengths were constrained by the limited size of the anatomic regions from which tissues were excised; for most scleral regions, the maximum excised tissue length obtainable was approximately 10 mm. Adhesive fixation of the specimen ends reduced the effective gauge length. Accordingly, the specimen lengths reported in Table 1 represent the initial gauge length measured between grips after mounting, rather than the total excised tissue length. Although anatomic constraints prevented attainment of very high length-to-width aspect ratios, the specimen aspect ratios used in this study lie within ranges previously evaluated for soft tissues, where changes in aspect ratio have been shown to exert relatively modest effects on measured mechanical properties compared with inherent biological variability.^32^

**Table 1.** Specimen Dimensions

Characteristic	Anterior Sclera	Equatorial Sclera	Posterior Sclera	Peripapillary Sclera	Optic Nerve Sheath	Optic Nerve
Number	112	112	99	31	59	16
Thickness (mm)	0.88 ± 0.13	0.88 ± 0.13	1.88 ± 0.14	1.46 ± 0.34	0.86 ± 0.14	
Width (mm)	2.31 ± 0.35	2.4 ± 0.5	2.6 ± 1.0	3.4 ± 0.4	2.2 ± 0.5	
Initial length (mm)	6.0 ± 1.5	7.0 ± 1.5	6.3 ± 1.4	4.2 ± 1.0	7.8 ± 2.1	12.1 ± 3.5
Diameter (mm)						3.3 ± 0.3

Values are presented as mean ± standard deviation.

### Experimental Procedure

Experiments were conducted using a custom load cell as previously described.^16^^,^^33^ The load cell included a linear motor with a quadrature optical position encoder having a 1-nm resolution and a 100-mm/s maximum speed, in series with a sensitive strain sensor (LSB200; FUTEK, Irvine, CA, USA) with a 5-mN resolution, coupled via a frictionless air bearing to a movable clamp within an environmental chamber containing a water bath to maintain saturated humidity and physiological temperature (37°C) as sensed by a thermocouple, regulating the closed-loop water bath heating system.^33^ To maintain tissue hydration, Ringer's lactate solution (B. Braun Medical) was continuously dripped onto the specimens. After securing specimens in the clamps, a small preload was applied to eliminate slack, ensuring that the tissue was taut at test initiation (*t* = 0). This preload did not exceed 5% of the maximum intended load. Initial specimen length was then recorded at preload, and the force registered by the sensor was set to zero. Initial loading strain was set at 0.1 s⁻¹ to ensure sufficiently rapid loading.

The experimental protocol comprised two sets of tests. First, linearity assessment of viscoelastic stress relaxation was conducted over a range of small strain levels, ε_*i*_ = 4%, 5%, and 6%, to confirm that viscoelasticity was linear within this range of strain. From among these strains, an appropriate constant strain magnitude ε_0_ could be selected to ensure linear viscoelastic behavior during the second test, stress relaxation under the application of this constant strain ε_0_.

Each experimental set incorporated two sequential stages, as illustrated in Figure 1B. Initially, six cycles of preconditioning were applied at a 5% engineering strain to stabilize the mechanical response of the tissue. This was immediately followed by a stress relaxation tensile test, in which the specimen was subjected to the predetermined constant strain ε_0_. The strain was maintained while the corresponding engineering stress relaxation data were continuously recorded until the tissue reached mechanical equilibrium. All experimental loading procedures were automated and executed via custom-developed software written in C++, which controlled the load cell system.

#### Determination of ε_0_ Using a Generalized Exponential Decay Model

To determine the constant strain ε_0_ to be employed for testing, a generalized one-phase Maxwell exponential decay model was fit to average uniaxial experimental stress relaxation data from a set of specimens.^34^ Curve fitting was performed using GraphPad Prism version 10.3.1 (GraphPad Software, La Jolla, CA, USA) for a series of constant applied strains: ε_*i*_ = 4%, 5%, and 6%.

Stress relaxation behavior was modeled using the following equation:

(1) $$\sigma \left(t\right)=\left(\sigma_{0}-\sigma_{\infty }\right)\exp\left(-\frac{t}{\tau }\;\right)+\sigma_{\infty }$$

where σ(*t*) is stress, σ_0_ is peak stress, σ_∞_ is equilibrium stress, *t* is time, and τ is characteristic time.

Characteristic time τ was extracted by curve fitting for each strain ε_*i*_ and plotted for all tested values. The slope of each regression line did not differ significantly from zero, indicating no systematic variation in the characteristic relaxation time with varying strain. The strain level at which τ remained approximately constant, indicating independence from strain amplitude, was identified as ε_0_. This selection is based on the principle that, within a small range of strain, for a material exhibiting linear viscoelastic behavior, the characteristic relaxation time τ should remain invariant with respect to changes in strain.^35^ The identified value of ε_0_ was then used in the second set of experiments, which involved stress relaxation testing under constant strain.

#### Determination of Prony Series Coefficients

To characterize the time-dependent viscoelastic properties of ocular tissues, a generalized Maxwell model and Prony series^36^ were used (an example of a second-order Prony series is illustrated in Fig. 1C).

In this model, the uniaxial relaxation stress is divided by ε_0_, and the relaxation modulus as a function of time is obtained as follows:

(2) $$E\left(t\right)=E_{\infty }+\sum_{n=1}^{N}E_{n}\exp\left(-\frac{t}{\tau_{n}}\;\right)$$

where *E*(*t*) denotes the relaxation modulus, *N* is the total number of spring-dashpot elements in the model, and *E_n_* represents the stiffness of the *n*th element, which decays with associated relaxation time τ_*n*_. The relaxation time $\tau_{n}=\frac{\eta_{n}}{E_{n}}$, where η_*n*_ is the viscosity of the *n*th dashpot component.

By rearranging Equation (2) and normalizing by *E*_0_, the instantaneous tensile modulus at *t* = 0, the dimensionless form of the relaxation modulus can be expressed as

(3) $$E\left(t\right)=E_{0}\left[1-\sum_{n=1}^{N}g_{n}\left\{1-\exp\left(-\frac{t}{\tau_{n}}\;\right)\right\}\right]$$

where $g_{n}=\frac{E_{n}}{E_{0}}$. Therefore, *g*(*t*) = *E*(*t*)/*E*_0_ is defined as the normalized relaxation modulus.

The relaxation modulus for each tissue specimen was determined by fitting a second-order Prony series using the Optimization Toolbox (lsqnonlin) in MATLAB R2024a (MathWorks, Natick, MA, USA). From these fits, the Prony coefficients *g_n_* and relaxation times τ_*n*_ were obtained. The instantaneous modulus *E*_0_ and equilibrium modulus *E*_∞_ were subsequently calculated from the fitted Prony parameters. The quality of agreement between experimental data and the Prony series model was quantified using the root mean squared error (RMSE), with lower values indicating a better fit.

### Statistical Analysis

Because multiple specimens were obtained from the same eye, and specimens were taken from both eyes of each donor, specimen-level measurements were not treated as statistically independent observations. For each anatomic region, Prony series parameters and derived moduli (instantaneous modulus *E*_0_ and equilibrium modulus *E*_∞_) were first averaged within each eye to obtain eye-level mean values. These eye-level averages were used for subsequent statistical analyses.

To account for possible between-eye correlations within subjects, statistical analysis of regional comparisons was performed using generalized estimating equations (GEEs, implemented in IBM SPSS, Chicago, IL, USA), which have type I error characteristics superior to those of *t*-tests for these samples.^37^ Model results are reported as estimated marginal means ± standard error (SE), and statistical significance was assessed using Wald χ^2^ tests with a threshold of *P* < 0.05.

## Results

### Linearity Test and Determination of ε_0_

**Figure 2.**
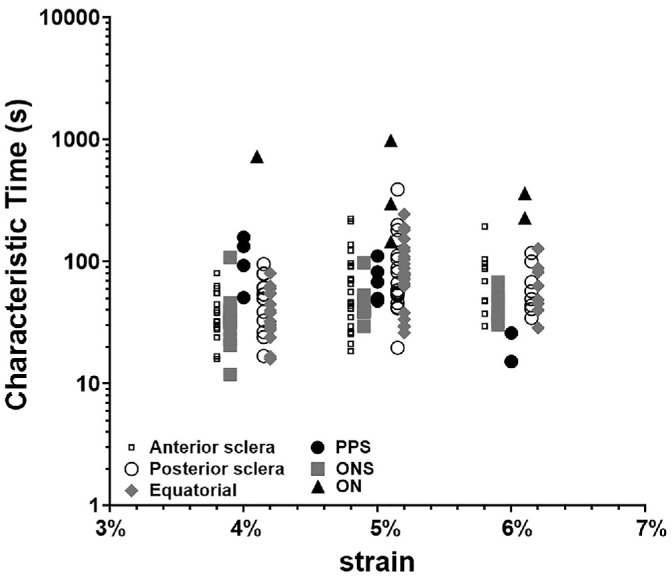
Linearity testing over a strain range of 4% to 6%. Each symbol represents a stress relaxation test for a single specimen. Data points have been slightly offset along the x-axis for clarity about discrete 4%, 5%, and 6% values.

To evaluate potential strain dependence, linear regression analyses were performed for each tissue type across the strain range from 4% to 6% using the log-transformed characteristic relaxation time as the dependent variable. For all tissues, regression slopes were not significantly nonzero (*P* > 0.05), indicating that no systematic trend in relaxation time with increasing strain was detectable within this range. Although substantial interspecimen variability was observed (Fig. 2), this variability did not correspond to a monotonic dependence on strain magnitude. These findings support the assumption of linear viscoelastic behavior within the tested strain range. Among the tested levels, an intermediate strain of 5% was selected for subsequent experiments due to its robust signal quality and relatively low variability.

### Stress Relaxation Results

**Figure 3.**
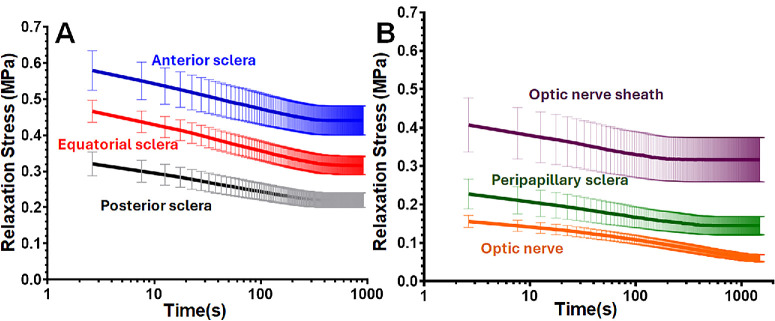
Average semi-logarithmic relaxation stress ± SE across all donors. (**A**) Stress for different regions of the sclera: anterior (*blue*), equatorial (*red*), and posterior (*black*). (**B**) Stress for the optic nerve sheath (*violet*), peripapillary sclera (*green*), and optic nerve (*orange*).

Stress relaxation responses were measured at an applied strain of *ε*₀ = 5% for anterior, equatorial, and posterior sclera (Fig. 3A), as well as peripapillary sclera, optic nerve sheath, and optic nerve (Fig. 3B). For each tissue region, stress–time curves represent the mean relaxation response ± SE, calculated from eye-level averaged data across all donors.

As shown in Figure 3A, the anterior sclera exhibited the highest relaxation stress throughout the test duration, followed by the equatorial and posterior sclera. In Figure 3B, the ONS showed higher stress levels than the PPS and ON, while the ON exhibited the least stress among all tissues examined. The slopes of the relaxation curves for the three scleral regions were similar, indicating comparable relaxation rates despite differences in stress magnitude. The optic nerve exhibited the longest characteristic relaxation time among all tissues tested.

**Figure 4.**
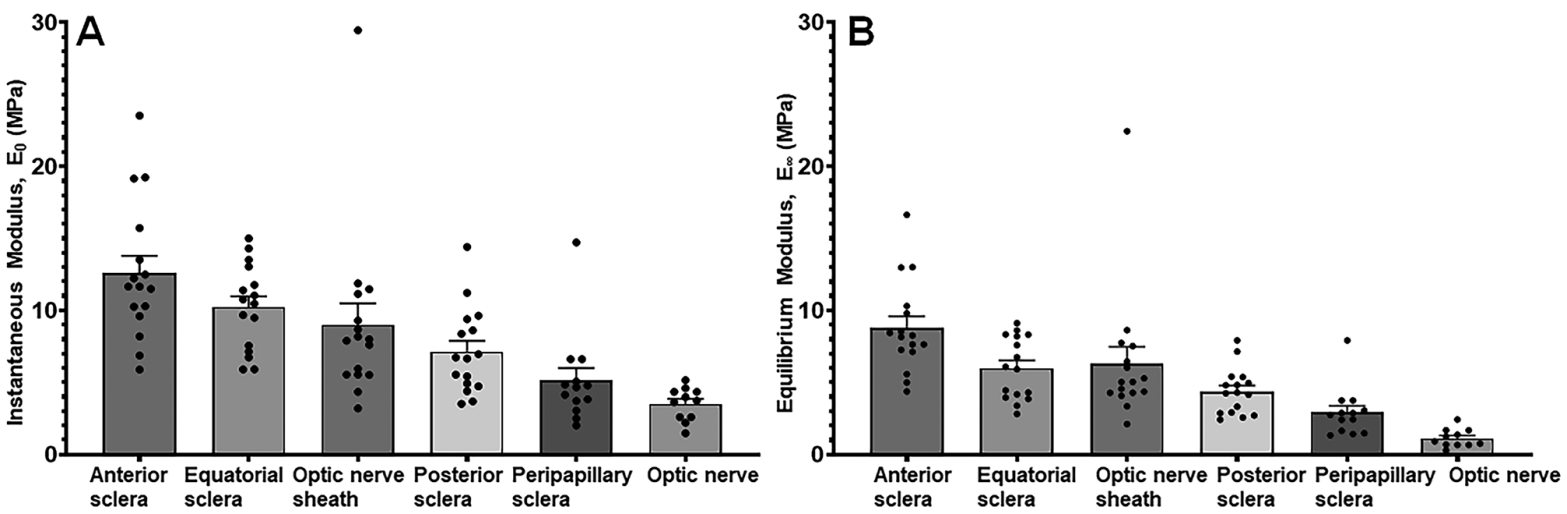
Regional variation in viscoelastic stiffness. (**A**) Instantaneous modulus (*E*_0_) and (**B**) equilibrium modulus (*E*_∞_) derived from second-order Prony series fits to stress relaxation data at ε₀ = 5%. *Bars* represent estimated marginal means ± SE. Overlaid points indicate eye-level means.

A comparison of the instantaneous modulus (*E*_0_) and equilibrium modulus (*E*_∞_) among tissues revealed significant regional heterogeneity in mechanical stiffness after accounting for repeated measurements within individual eyes using GEE (Fig. 4). For both moduli, stiffness decreased systematically from anterior-to-posterior connective tissues and was substantially lower in neural tissues.

For the instantaneous modulus, GEE analysis demonstrated a significant main effect of tissue region (Wald χ^2^, *P* < 0.001). Estimated marginal means showed that the anterior sclera had the highest mean stiffness *E*_0_ at 12.6 ± 1.2 MPa (SE), followed by equatorial sclera at 10.2 ± 0.7 MPa and posterior sclera at 7.1 ± 0.7 MPa. The ONS demonstrated intermediate stiffness at 9.0 ± 1.5 MPa, whereas PPS had a lower stiffness of 5.1 ± 0.9 MPa. The ON was most compliant, with still lower instantaneous modulus at 3.5 ± 0.3 MPa. Multivariable models demonstrated no significant effects of age, sex, or eye laterality for *E*₀, indicating that regional differences in instantaneous stiffness were driven primarily by tissue-specific properties.

For equilibrium modulus, GEE analysis again revealed a significant effect of tissue region (Wald χ² = 19.9, *df* = 5, *P* = 0.001), as well as a significant interaction of tissue and age (Wald χ² = 22.3, *df* = 5, *P* < 0.001). This suggests indicating that age-related changes in long-term stiffness differ across tissues. However, main effects of age, sex, and eye laterality were not significant. Estimated marginal means demonstrated that the anterior sclera again exhibited the highest *E*_∞_ at 8.8 ± 0.8 MPa, followed by the equatorial sclera at 6.0 ± 0.5 MPa and posterior sclera at 4.4 ± 0.4 MPa. The ONS showed intermediate equilibrium stiffness at 6.3 ± 1.1 MPa, while the PPS was less at 2.9 ± 0.5 MPa. The ON had the least equilibrium modulus at 1.1 ± 0.2 MPa, approximately eightfold less than the anterior sclera.

Tissue-dependent differences in the Prony series parameters describing viscoelastic relaxation behavior were revealed by GEE analysis (Table 2). Both the short-term (*g*₁) and long-term (*g*₂) relaxation magnitudes differed significantly across tissues (*P* < 0.01). Scleral regions exhibited relatively similar *g*_1_ values, whereas the ON showed the largest short-term relaxation magnitude, indicating a greater contribution of rapid viscoelastic processes. For *g*_2_, there was a significant main effect of tissue (Wald χ² = 19.6, *df* = 5, *P* = 0.001) and a significant interaction of tissue and age (Wald χ² = 15.5, *df* = 5, *P* = 0.009), demonstrating that age-related changes in long-term relaxation magnitude differed by tissue region. This interaction was driven primarily by the ON, which showed an age-dependent increase in *g*_2_,  whereas age effects were negligible in the sclera. Significant tissue-dependent differences were also observed in the characteristic relaxation times. The short relaxation time constant τ_1_ varied significantly across tissues (Wald χ² = 29.0, *df* = 5, *P* < 0.001), with a significant interaction of age and tissue (Wald χ² = 41.9, *df* = 5, *P* < 0.001). The long relaxation time constant τ_2_ exhibited strong tissue dependence (*P* < 0.001) and a significant interaction of tissue and age (*P* < 0.001). The ON exhibited markedly longer relaxation time (τ_2_ = 460 ± 77 seconds) than all other tissues, whereas the ONS had the shortest relaxation time (τ_2_ = 60 ± 5 seconds). In contrast, scleral regions displayed intermediate and similar relaxation times, suggesting comparable rates of stress decay despite pronounced differences in regional scleral stiffness.

**Table 2.** Prony Series Parameters for Stress Relaxation

Tissue	*g*1	*g*2	τ_1_ (s)	τ_2_ (s)	RMSE (MPa)
Anterior sclera	0.15 ± 0.01	0.16 ± 0.01	4.3 ± 0.3	93 ± 5	0.05 ± 0.01
Equatorial sclera	0.21 ± 0.02	0.23 ± 0.02	4.5 ± 0.4	112 ± 12	0.06 ± 0.01
Posterior sclera	0.18 ± 0.02	0.21 ± 0.02	4.2 ± 0.4	105 ± 13	0.04 ± 0.01
Peripapillary sclera	0.21 ± 0.03	0.22 ± 0.02	5.8 ± 0.6	115 ± 9	0.02 ± 0.01
Optic nerve sheath	0.14 ± 0.01	0.16 ± 0.02	3.1 ± 0.3	60 ± 5	0.03 ± 0.01
Optic nerve	0.29 ± 0.03	0.41 ± 0.04	13 ± 2	460 ± 77	0.03 ± 0.01

Values are presented as mean ± standard deviation of Prony coefficients (*g*_1_, *g*_2_) and relaxation time constants (τ_1_, τ_2_) obtained from second-order fits to stress relaxation data at ε_0_ = 5%.

Across all tissues, RMSE values were low (typically 0.02–0.06 MPa), indicating good agreement between the Prony series model and experimental data.

## Discussion

This study quantified the viscoelastic behavior of human ocular tissues during stress relaxation and demonstrated pronounced regional differences in mechanical properties across the eye. Linear viscoelastic behavior was observed within the tested strain range (4%–6%), encompassing the anterior, equatorial, and posterior sclera; PPS; optic nerve sheath; and ON. These findings are consistent with prior reports of approximately linear viscoelastic responses in ocular tissues under low-strain creep and stress–relaxation loading.^16^^,^^31^^,^^38^ Previous studies have shown that nonlinearity and strain-dependent effects emerge primarily at higher strain magnitudes.^39^ Although the present analysis was confined to a narrow strain range, similar Prony series–based relaxation kernels have been shown to integrate naturally within quasi-linear viscoelastic formulations at finite strains.^19^ Accordingly, the relaxation time constants and relative moduli identified here may remain relevant within nonlinear elastic frameworks, even as instantaneous stress–strain behavior departs from linearity at higher strains.^40^

Marked regional differences in stress relaxation behavior were observed, reflecting distinct biomechanical roles. The anterior sclera and ON exhibited strongly contrasting responses, with the anterior sclera displaying high stiffness and limited stress decay, and the ON exhibiting pronounced compliance and slow relaxation. These differences are consistent with the functional requirement of the anterior sclera to preserve globe shape under loading,^41^ while the ON must remain compliant to mitigate traction and deformation during eye movements.^42^^,^^43^ Such spatial heterogeneity in viscoelastic behavior may influence local stress transmission and deformation patterns, with implications for posterior eye disorders such as optic neuropathy and myopia.

Under a constant 5% strain, the anterior sclera exhibited the smallest reduction in stress over time among scleral regions, whereas the ON demonstrated the greatest reduction. This behavior is quantified by the instantaneous and equilibrium moduli: the anterior sclera exhibited the highest values, whereas the ON was substantially lower. These instantaneous moduli are consistent in both magnitude and regional trends with prior experimental measurements of scleral elastic properties summarized by Boote et al.,^4^ who report tensile and inflation-based elastic moduli spanning several to tens of megapascals, with anterior regions stiffer than posterior regions. The anterior-to-posterior decrease in stiffness observed here reproduces this established gradient. Differences in absolute modulus values across studies are expected given the strong dependence of measured stiffness on testing modality, strain magnitude, fiber orientation, and postmortem conditions. These results also align with prior studies reporting higher stiffness in anterior sclera^16^^,^^27^ and lower stiffness in the ON, possibly due to the presence of internal fluid within the ON^28^ or differences in fibrillar organization.^44^ Notably, the ONS was stiffer than the ON under similar imposed strain, supporting previous suggestions that the sheath bears a greater mechanical load during eye movements.^42^ These distinctions underscore the importance of incorporating tissue-specific viscoelastic parameters into biomechanical models of the eye.

The regional variation in scleral stiffness is consistent with known differences in collagen architecture. The anterior sclera has higher collagen density and a more interwoven fiber network,^4^^,^^45^ whereas the posterior sclera shows a more lamellar organization associated with lower stiffness.^4^ The PPS is highly anisotropic, with collagen fibers preferentially aligned circumferentially around the ON head.^46^^,^^47^ However, wide-angle X-ray scattering studies indicate that this highly aligned circumferential collagen ring is confined to a narrow region immediately surrounding the ON head.^30^ Specimens tested in this study probably included less isotropic tissue peripheral to the main circumferential ring, so the values reported here probably yielded lower effective stiffness and instantaneous moduli.^4^^,^^46^

Analysis of Prony series parameters clarifies tissue-specific viscoelastic behavior. Both short-term and long-term relaxation magnitudes and relaxation time constants differed significantly across tissues, indicating distinct contributions of fast and slow viscoelastic processes. Neural tissues, particularly the ON, exhibited larger long-term relaxation magnitudes and markedly longer relaxation times, reflecting slow stress decay and enhanced time-dependent deformation. In contrast, scleral tissues displayed intermediate and relatively similar relaxation times, suggesting comparable rates of stress decay despite large differences in stiffness. Age-dependent effects were tissue-specific, with significant tissue-by-age interactions observed for several parameters, indicating that aging influences viscoelastic behavior differently across ocular tissues rather than producing a uniform global effect. Although RMSE values were uniformly small across tissues, indicating good model agreement, variability in relaxation time constants, particularly for the ON, likely reflects reduced sensitivity of stress relaxation data to late-time behavior, a known limitation of viscoelastic parameter identification.

Despite these insights, the current study has some limitations. Uniaxial testing of specimens excised from curved ocular tissues introduces inherent geometric simplifications, as curvature can lead to nonuniform strain distributions and initial strains associated with specimen flattening, as previously discussed for corneal strip extensometry.^48^ There is no straightforward way to avoid this limitation. Specimen geometry in the present study was further constrained by the limited size of ocular anatomic regions, and specimen dimensions therefore could not be based on standardized uniaxial testing geometries. Specimen thickness was measured using digital calipers, but because ocular tissues are thin, compliant, and subject to hydration, measurements represent an effective thickness and may exceed values reported using alternative measurement approaches.^27^^,^^49^ However, all measurements were conducted within the linear viscoelastic range (4%–6% strain), and no visible macroscopic necking or strain localization was observed. At higher strain levels, tissue response may become nonlinear, requiring more complex formulations beyond standard linear models.^39^

The assumption that ocular tissues, particularly the ON, behave purely viscoelastically may not fully capture their mechanical complexity, because internal fluid redistribution can contribute to deformation.^29^ Both sclera and ON have therefore been modeled as poroelastic materials,^50^^,^^51^ and future studies could investigate combined poro-viscoelastic behavior.
